# Serum Adiponectin Predicts COVID-19 Severity

**DOI:** 10.3390/biomedicines12051043

**Published:** 2024-05-09

**Authors:** Vlad Pavel, Ulrich Räth, Stephan Schmid, Sabrina Krautbauer, Dennis Keller, Pablo Amend, Martina Müller, Patricia Mester, Christa Buechler

**Affiliations:** 1 Department of Internal Medicine I, Gastroenterology, Hepatology, Endocrinology, Rheumatology, and Infectious Diseases, University Hospital Regensburg, 93053 Regensburg, Germany; vlad.pavel@klinik.uni-regensburg.de (V.P.); ulrich.raeth@stud.uni-regensburg.de (U.R.); stephan.schmid@klinik.uni-regensburg.de (S.S.); dennis.keller@stud.uni-regensburg.de (D.K.); pablo.amend@stud.uni-regensburg.de (P.A.); martina.mueller-schilling@klinik.uni-regensburg.de (M.M.); patricia.mester@klinik.uni-regensburg.de (P.M.); 2 Institute of Clinical Chemistry and Laboratory Medicine, University Hospital Regensburg, 93053 Regensburg, Germany; sabrina.krautbauer@klinik.uni-regensburg.de

**Keywords:** adiponectin, disease severity, C-reactive protein, intensive care, bacterial superinfection, COVID-19

## Abstract

Adiponectin is primarily known for its protective role in metabolic diseases, and it also possesses immunoregulatory properties. Elevated levels of adiponectin have been observed in various inflammatory diseases. However, studies investigating adiponectin levels in the serum of COVID-19 patients have yielded conflicting results. This study aimed to assess serum adiponectin levels in 26 healthy controls, as well as in 64 patients with moderate and 60 patients with severe COVID-19, to determine a potential association between serum adiponectin and the severity of COVID-19. Serum adiponectin levels in severe COVID-19 patients were significantly lower than in those with moderate disease and healthy controls, who exhibited similar serum adiponectin levels. Among patients with moderate disease, positive correlations were observed between serum adiponectin and C-reactive protein levels. Of note, serum adiponectin levels of severe COVID-19 cases were comparable between patients with and without dialysis or vasopressor therapy. Superinfection with bacteria did not exert a notable influence on serum adiponectin levels in patients with severe disease. Patients who were diagnosed with severe COVID-19 and vancomycin-resistant enterococci bacteremia showed a significant reduction in their serum adiponectin levels. An analysis conducted on the entire cohort, including both moderate and severe COVID-19 patients, showed that individuals who did not survive had lower serum adiponectin levels when compared to those who survived. In summary, this study highlights a decrease in serum adiponectin levels in severe COVID-19 cases, indicating the potential utility of adiponectin as an additional biomarker for monitoring disease severity in COVID-19 or critical illnesses in general.

## 1. Introduction

Coronavirus disease 2019 (COVID-19), caused by infection with severe acute respiratory syndrome coronavirus type 2 (SARS-CoV-2), has very heterogeneous symptoms ranging from asymptomatic infections to lethal outcomes [1]. As is the case with other viral infections, SARS-CoV-2 triggers an innate immune reaction by recruiting neutrophils, monocytes, and macrophages, and releasing cytokines, which prime lymphocytes for an adaptive immune response. This successfully clears the infection for approximately 80% of cases. However, an overwhelming release of pro-inflammatory cytokines such as interleukin (IL)-1 and IL-6 in some patients can cause severe tissue injury that requires intensive care in hospitals [2].

Obesity and metabolic disorders related to obesity are common factors associated with more severe COVID-19 disease, and low-grade inflammation associated with these diseases is suggested to contribute to the higher risk [3]. 

Serum levels of most of the multiple adipokines studied so far increase in obesity, partly driven by a higher synthesis in adipose tissues [4,5,6]. Adiponectin is one of the few exceptions, and fat cell production as well as circulating levels of adiponectin decline in obesity [5,7,8]. Adiponectin is a very well-studied adipokine, and was shown to improve insulin response, type 2 diabetes, liver function, and cardiovascular diseases [5,8]. Inflammatory cytokines reduce adiponectin release from adipose tissues and patients with viral infections have low serum levels of adiponectin [9]. 

SARS-CoV-2 infection of adipocytes and adipose tissue macrophages has been described to induce a local inflammatory response [9]. Adiponectin is almost exclusively produced by adipocytes, and infection of these cells with SARS-CoV-2 reduces adiponectin synthesis [8,10,11]. As there is evidence that adiponectin acts as an anti-inflammatory factor, suppression of adiponectin may drive inflammation in COVID-19 [5,7,12]. The severity of COVID-19 is significantly influenced by the involvement of oxidative stress [13]. Adiponectin not only combats inflammation but also confronts oxidative stress. Several studies have showcased its capacity to reduce the generation of reactive oxygen species while activating antioxidant pathways [14,15,16,17]. 

The relationship between serum adiponectin levels, SARS-CoV-2 infection, and COVID-19 severity is still unclear. A comparison of COVID-19 patients and healthy controls revealed lower serum adiponectin levels in the patients. There was also evidence for a further decline in serum adiponectin in severely ill COVID-19 patients [18,19,20,21]. Other investigations could not detect reduced adiponectin levels in COVID-19 or associations with disease severity [22,23,24]. An additional study described higher serum adiponectin levels in COVID-19 patients compared to healthy controls [25].

Studies have also compared patients who were similarly ill, with and without SARS-CoV-2 infection. One of these investigations revealed higher levels of IL-4, IL-5, and IL-6 in patients with COVID-19 pneumonia compared to those suffering from bacterial community-acquired pneumonia, while adiponectin levels showed no significant differences between these two groups [26]. These findings are consistent with results from two other studies [18,27]. Consequently, it is plausible that circulating adiponectin levels decrease in critical illness, regardless of SARS-CoV-2 infection [28]. However, it is worth noting that patients with COVID-19-related respiratory failure exhibited lower plasma adiponectin levels when compared to patients with respiratory failure unrelated to this virus [19].

In summary, studies that have investigated serum adiponectin levels in COVID-19 patients yielded inconsistent results regarding associations with disease severity and mortality [28]. The aim of this study was to evaluate the relationship between serum adiponectin levels and COVID-19 disease severity, adjusting for factors known to influence circulating adiponectin levels, such as body mass index (BMI) and diabetes [8,29]. The prevalence of liver cirrhosis in Germany is almost 1% [30] and these patients have highly elevated serum adiponectin [31], which was taken into account in this analysis. This study also shows that liver cirrhosis is associated with lower levels of IL-6, a cytokine often measured to assess COVID-19 severity [32]. Furthermore, in contrast to some previous studies [28], the associations of serum adiponectin were performed separately in patients with moderate and patients with severe COVID-19 to identify relationships of serum adiponectin with bacterial infections and mortality independent of disease severity. 

## 2. Materials and Methods

### 2.1. Study Cohort

**Figure 1:**
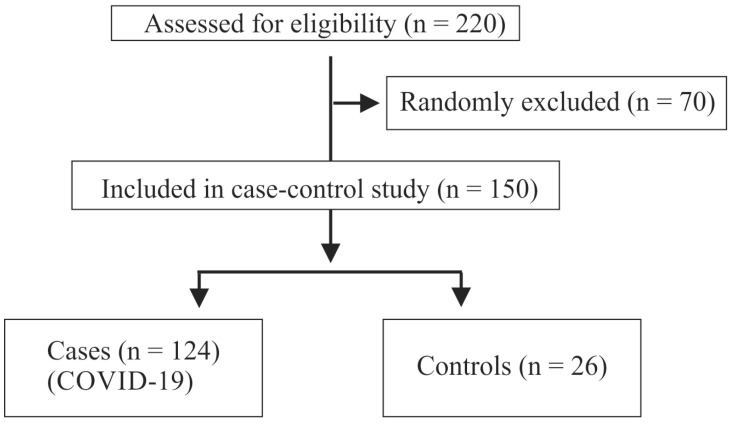
Consort flow diagram of case–control study.

Blood samples of 194 patients were collected from patients aged 18 years or older between 16 April 2020 and 12 January 2024 at the University Hospital of Regensburg. SARS-CoV-2 infection was confirmed by polymerase chain reaction. All patients were hospitalized for COVID-19 disease. For the current analysis, 124 samples were randomly selected (Figure 1). 

Moderate disease was characterized as the presence of systemic inflammatory response syndrome (SIRS) without the need for intensive care [33,34], while severe COVID-19 was defined as patients experiencing sepsis or septic shock [33,34]. Blood samples of our moderate patients were collected 3 (1–16) days after hospital admission, and those of our patients with severe COVID-19 were collected 4 (1–10) days after hospital admission. This shows that the patients had already received COVID-19 therapy at the time of blood collection. In the severe cohort, serum CRP (*p* = 0.641) and procalcitonin (*p* = 0.981) did not correlate with the day of blood collection, meaning that day of blood collection is not related to these measures of disease severity. 

As 64 patients presented with dyspnea, tachycardia, fever, and fatigue, and fulfilled the criteria for systemic inflammatory response syndrome (SIRS), they were included in the moderate COVID-19 group [33]. Notably, our SIRS group is clinically equivalent to moderate COVID-19 according to the National Institutes of Health COVID-19 severity classification [35]. These patients were admitted to the hospital for monitoring but did not require admission to intensive care. Sixty patients were treated in the intensive care unit because most of them developed acute respiratory distress syndrome (ARDS) and septic shock. Our severe COVID-19 group corresponds to critical illness according to the National Institutes of Health classification of COVID-19 severity [34,35,36,37]. 

The Horowitz index (also known as the partial arterial pressure of oxygen (PaO2, mmHg)/fraction of inspired oxygen index) was calculated for all COVID-19 patients receiving invasive ventilation in the intensive care unit. An index below 200 mmHg was defined as ARDS. The median Horowitz index of our patients was 147 mmHg (range 21–302).

Common confounding factors such as hypertension or diabetes were documented. Rare (less than 3 patients) comorbidities in the moderate COVID-19 were Morbus Wilson, low phospholipid associated cholelithiasis, chronic kidney insufficiency, post liver transplant, heart valvular insufficiency, post heart transplant, autoimmune hepatitis, Morbus Crohn, anemia, pulmonary embolism, multiple myeloma, and chronic lymphatic leukemia. In the severe group rare comorbidities were asthma, chronic obstructive pulmonary disease, hyperuricemia, pancreatitis, secondary sclerosing cholangitis, and Churg Strauss Disease. 

The COVID-19 patients were treated according to the current COVID-19 guidelines as approved by the European Medicines Agency and the German Federal Joint Committee. Most of the blood samples were collected in 2021, and at that time, the only drugs approved for the treatment of COVID-19 in Germany were remdesivir and dexamethasone. These drugs were also used for the few patients from whom serum was collected in 2024. 

To prevent thrombosis, all patients received either low-molecular-weight heparin or unfractionated heparin. Drugs targeting the cytokine release syndrome in COVID-19, such as tocilizumab, baricitinib, or sotrovimab, were not approved in Germany at that time and were not given to any patient. 

Serum from 64 patients with moderate COVID-19, 60 patients with severe COVID-19, and 26 age- and sex-matched controls were analyzed in this study (Figure 1). The 26 controls were healthy hospital staff and relatives of the medical students involved in the project. Here, preferentially older and male individuals with normal BMI were asked to participate in the study.

The study was conducted following the Declaration of Helsinki and approved by the Ethics Committee of the University Hospital of Regensburg (protocol code 18-1029_2-101, 14.03.2023). Informed consent was obtained from all subjects involved in the study. 

### 2.2. Adiponectin ELISA

The human adiponectin DuoSet ELISA (R&D Systems; Wiesbaden, Nordenstadt, Germany) was used as recommended by the manufacturer (dilution of serum was 1:5000). Serum levels of adiponectin were measured in duplicate and the mean adiponectin values were used for calculations. 

### 2.3. Analysis of Laboratory Measures

The levels of C-reactive protein (CRP) were evaluated using an enhanced method for immunoturbidimetric assays. Procalcitonin and IL-6 levels were measured utilizing ElektroChemiLumineszenz ImmunoAssays. The conversion of L-lactate to pyruvate, catalyzed by lactate dehydrogenase (LDH), leads to the reduction of NAD+ to NADH, which can be directly determined photometrically. Similarly, lactate oxidase and hydrogen peroxide facilitate the oxidation of L-lactate to pyruvate, resulting in the production of a dye via peroxidase. This dye’s intensity is then assessed photometrically. Alkaline phosphatase (AP) activity is determined based on the hydrolysis reaction involving p-nitrophenyl phosphate into phosphate and p-nitrophenol, both measurable through photometry. Ferritin measurement involves an ElektroChemiLumineszenz ImmunoAssay that employs ferritin-specific monoclonal antibodies linked with either ruthenium or biotin as conjugates. Lipoprotein lipase breaks down triglycerides into glycerol and fatty acids; their measurement requires subsequent oxidation to dihydroacetone phosphate and hydrogen peroxide, which produces a red dye when 4-aminophenazone and 4-chlorphenol are introduced. All these tests were conducted using a Cobas Pro analyzer along with corresponding Roche assays from Penzberg, Germany. 

Differential blood count was obtained using the impedance/flow cytometry methodology implemented with a Sysmex instrument provided by Sysmex Deutschland GmbH located in Bornbarch, Germany. Analysis for all laboratory parameters mentioned above was performed at the Institute of Clinical Chemistry and Laboratory Medicine at University Hospital Regensburg. Enterococci resistance to vancomycin was confirmed by analysis of the genes van A and/or van B by PCR at the Institute of Clinical Microbiology and Hygiene, University Hospital Regensburg, Regensburg, Germany. 

### 2.4. Statistical Analysis

A power analysis (G*Power 3.1.6) using data of Perrotta et al. [20] with alpha = 0.05 and power of 0.95 revealed that 17 controls and 33 patients had to be included. This shows that our cohort was large enough. 

Boxplots depict key statistics including minimum and maximum adiponectin values, the median, and the first and third quartiles. Outliers are represented as individual asterisks (extreme outliers) or circles (mild outliers). Tables provide the data in terms of median, minimum, and maximum values. Statistical analysis was conducted using non-parametric tests, specifically the Mann–Whitney U-test to compare two independent groups, the Kruskal–Wallis test to compare three independent groups, and Spearman’s correlation, which is a nonparametric correlation. Categorical variables were compared using the chi-square Test. The receiver operating characteristic curve was used to determine the discriminatory ability of laboratory measures for survival, and multiple linear regression analysis was used to predict non-survival and disease severity. All calculations were performed using IBM SPSS Statistics 26.0 (Chicago, IL, USA). A significance level of *p* < 0.05 was considered significant.

## 3. Results

### 3.1. Serum Adiponectin Levels of Controls and COVID-19 Patients 

This study analyzed the serum of 64 patients with moderate COVID-19, 60 patients with severe COVID-19 requiring intensive care treatment, and 26 sex- and age-matched controls. The common comorbidities of hypertension and cardiovascular disease were more often diagnosed in moderate COVID-19 patients, and diabetes prevalence was similar between the patient groups (Table 1). 

Patients with severe COVID-19 had higher CRP, procalcitonin, ferritin, and LDH levels in comparison to patients with moderate disease (Table 1). Neutrophils, basophils, monocytes, and immature granulocytes in blood were increased (Table 1). BMI of patients with severe COVID-19 was higher, whereas age, sex distribution, AP, IL-6, and lactate were similar among the two groups (Table 1). Controls were matched for age and sex, and BMI of controls and patients with moderate COVID-19 was similar (Table 1). 

**Figure 2:**
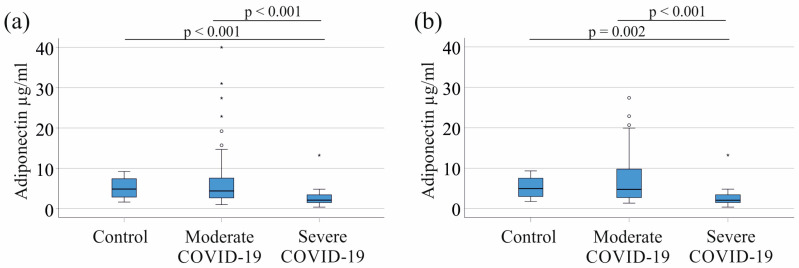
Serum adiponectin levels of controls, and moderate and severe COVID-19 patients: (**a**) patients with liver cirrhosis are included; (**b**) patients with liver cirrhosis are excluded. Outliers are represented as individual asterisks (extreme outliers) or circles (mild outliers).

Adiponectin serum levels of controls and patients with moderate disease were similar (*p* = 0.073). Serum levels of patients with severe COVID-19 were reduced in contrast to controls (*p* < 0.001) and in comparison to patients with moderate disease (*p* < 0.001) (Table 1 and Figure 2a). 

### 3.2. Adiponectin in Relation to Age, Sex, Adiposity, Comorbidities and Liver Cirrhosis

Among the moderate COVID-19 group, four patients were found to have liver cirrhosis. In the severe COVID-19 cohort, only one patient was identified with liver cirrhosis. 

The serum adiponectin level in patients without liver cirrhosis was 3.1 (range: 0.4–27.4) µg/mL, whereas those with cirrhosis recorded a level of 19.2 (range: 2.7–40) µg/mL, resulting in significantly higher serum adiponectin levels compared to COVID-19 patients without liver cirrhosis (*p* = 0.001). 

Due to the elevated serum adiponectin levels observed in patients with liver cirrhosis, we excluded five patients from subsequent analyses. Of note, this exclusion had no impact on the distribution of serum adiponectin levels among the control group, patients with moderate COVID-19, or those with severe COVID-19. Notably, serum adiponectin levels in the control group were comparable to those in patients with moderate disease (*p* = 0.128), whereas patients with severe COVID-19 exhibited significantly lower levels compared to both the control group (*p* = 0.002) and patients with moderate disease (*p* < 0.001) (Figure 2b). Adiponectin, CRP, and procalcitonin predicted severe COVID-19 *F*(3, 98) = 13.12, *p* < 0.001) better than CRP and PCT (*F*(2, 99) = 4.20, *p* = 0.018) alone. 

It should be noted that patients with liver cirrhosis had a trend of higher serum IL-6 (*p* = 0.086). Exclusion of liver cirrhosis patients showed that serum IL-6 of moderate cases was 18.2 (4.0–265) pg/mL and of severe cases was 35.2 (3.0–1175) pg/mL, and was higher in the latter cohort (*p* = 0.049). 

**Figure 3:**
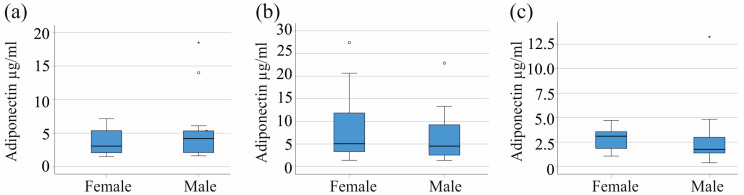
Serum adiponectin levels of males and females: (**a**) in the control cohort; (**b**) of patients with moderate COVID-19; and (**c**) of patients with severe COVID-19. Patients with liver cirrhosis were excluded. Outliers are represented as individual asterisks (extreme outliers) or circles (mild outliers).

Serum adiponectin levels between sexes in the control cohort (*p* = 0.385), the moderate COVID-19 group (*p* = 0.272), and the severe COVID-19 group (*p* = 0.068) exhibited no significant differences (Figure 3a–c).

Adiponectin showed no correlation with patient age in the control cohort (r = 0.124, *p* = 0.583) and the moderate COVID-19 group (r = 0.144, *p* = 0.277). However, a positive association with age was observed in the severe COVID-19 cohort (r = 0.273, *p* = 0.037). Regarding BMI, a negative correlation was found in the moderate cohort (r = −0.481, *p* = 0.010), whereas no significant correlation was detected in controls (r = −0.272, *p* = 0.198) and severely ill patients (r = −0.052, *p* = 0.707).

In the severe COVID-19 cohort, 16 patients had a BMI of ≥35 kg/m^2^, yet their serum adiponectin levels were similar to those of non-obese patients (*p* = 0.413). Among the obese patients in the severe COVID-19 cohort, ten were females, and they exhibited serum adiponectin levels comparable to those of non-obese females with severe COVID-19 (*p* = 0.626). The six obese men had similar serum adiponectin levels to the non-obese men (*p* = 0.122). When obese patients were excluded from the severe sepsis group, the eight females in this subgroup displayed serum adiponectin levels similar to those of the 35 males (*p* = 0.374).

Within the moderate COVID-19 group, only three patients had a BMI of ≥35 kg/m^2^, and after excluding patients with adiposity, there were no significant differences between sexes in terms of serum adiponectin levels (*p* = 0.986).

Diabetes (*p* = 0.916), cardiovascular disease (*p* = 0.781), and hypertension (*p* = 0.297) in the moderate COVID-19 group, and diabetes in the severe cohort (*p* = 0.598), were not associated with a change in serum adiponectin levels. 

### 3.3. Adiponectin in Relation to Interventions and Vasopressor Therapy 

Patients with moderate COVID-19 requiring dialysis tended to have higher serum adiponectin. Serum adiponectin levels did not show associations with the requirement for dialysis among severe COVID-19 patients (Table 2). In the severe COVID-19 group, all patients except one were invasively intubated, while none in the moderate group required invasive ventilation, making statistical analysis unfeasible. Among the 40 patients receiving vasopressor therapy in the severe COVID-19 cohort, their adiponectin levels were similar to those of patients not receiving these medications (Table 2).

### 3.4. Correlation of Serum Adiponectin Levels with Laboratory Measures and Inflammation Markers 

In patients with moderate disease, serum adiponectin positively correlated with CRP, procalcitonin, and neutrophil count (Table 3). In severe COVID-19, no such associations could be detected. Of note, in this cohort, serum adiponectin was negatively correlated with triglyceride levels (Table 3). 

### 3.5. Effect of Bacterial Infections on Serum Adiponectin Levels 

In the group of patients with moderate COVID-19, six patients had bacterial infection, which was not related to a change in serum adiponectin (*p* = 0.315)

**Figure 4:**
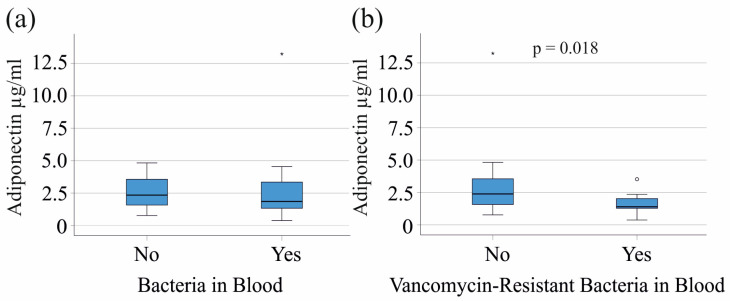
Adiponectin and bacterial superinfections of patients with COVID-19: (**a**) serum adiponectin of patients with severe COVID-19 without and with bacterial blood superinfections; (**b**) serum adiponectin of patients with severe COVID-19 without and with vancomycin-resistant superinfections. Patients with liver cirrhosis were excluded. Outliers are represented as individual asterisks (extreme outliers) or circles (mild outliers).

In contrast, among patients with severe COVID-19, 26 patients had bacterial infections. Adiponectin levels did not show significant differences between these groups (*p* = 0.285) (Figure 4a).

However, the 10 patients with severe COVID-19 and vancomycin-resistant bacteria had reduced serum adiponectin in comparison to severe cases without these bacteria (Figure 4b). Infected patients had lower BMI (*p* = 0.028) whereas CRP, procalcitonin, and leukocyte numbers were similar between the groups. 

### 3.6. Plasma Adiponectin and Survival

One patient died in the moderate disease cohort. The cause of death was respiratory failure due to COVID-19 pneumonia. The patient also had acute myeloid leukemia. This patient developed fulminant acute myeloid leukemia and died due to leukemia-related respiratory involvement and COVID-19.

**Figure 5:**
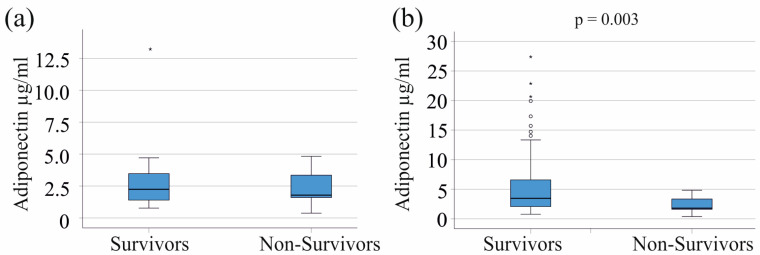
Adiponectin serum levels of survivors and non-survivors: (**a**) adiponectin serum levels of survivors and non-survivors in the severe COVID-19 cohort; (**b**) adiponectin serum levels of survivors and non-survivors in the entire COVID-19 cohort. Patients with liver cirrhosis were excluded. Outliers are represented as individual asterisks (extreme outliers) or circles (mild outliers).

In the severe COVID-19 group, 21 patients did not survive their severe illness. In patients with severe COVID-19, serum adiponectin levels did not differ between survivors and non-survivors (*p* = 0.837) (Figure 5a). An analysis conducted on the entire cohort, including both moderate and severe COVID-19 patients, showed that individuals who did not survive had lower serum adiponectin levels when compared to those who survived (*p* = 0.003) (Figure 5b).

In the severe COVID-19 group, non-survivors had higher procalcitonin (*p* = 0.007), neutrophil (*p* < 0.001), and immature granulocyte counts (*p* = 0.004). CRP, ferritin, IL-6, basophils, eosinophils, monocytes, and lymphocytes were not related to death. The area under the receiver operating characteristics curve to predict non-survival was 0.712 (*p* = 0.007), 0.779 (*p* < 0.001), and 0.726 (*p* = 0.004) for procalcitonin, neutrophils, and immature granulocytes, respectively. Procalcitonin, neutrophil, and immature granulocyte numbers were able to significantly predict non-survival in severe COVID-19, *F*(3, 55) = 6.28, *p* = 0.001.

## 4. Discussion

This study showed that serum adiponectin levels are lower in severe COVID-19 patients compared to controls and those patients with moderate disease. Notably, the latter two groups exhibited similar serum adiponectin levels, indicating that serum adiponectin levels are associated with COVID-19 disease severity. The adiponectin/leptin ratio was found to be induced in patients with moderate COVID-19 [9,23], suggesting unbalanced levels of these adipokines may play a role in patients with moderate and severe COVID-19. 

In severe COVID-19 cases, serum adiponectin levels did not exhibit a significant association with survival outcomes. However, when this analysis was extended to the entire cohort, it was found that non-survivors had lower serum adiponectin levels. Therefore, the relationship between serum adiponectin and mortality appears to be influenced by the inclusion of less severely ill COVID-19 patients, who had higher serum adiponectin levels and a lower mortality rate. This finding aligns with previous studies that also did not observe a significant association between systemic adiponectin levels and survival [18,23]. Flikweert et al. found no association between survival and the plasma adiponectin levels in the 71 critical COVID-19 patients included in their study. The mortality rate of our severe COVID-19 patients was 36% and that of the cohort studied by Flikweert et al. was nearly 44% [18]. Di Filippo et al. included 60 patients, 24 of whom required intensive care. The adiponectin/leptin ratio, but not adiponectin levels, was associated with mortality, with lower mortality in patients with a high adiponectin/leptin ratio [23]. Adiponectin may be regarded as a marker of disease outcome, which is dependent on disease severity. 

The status of serum adiponectin levels in SARS-CoV-2 infection remains uncertain. The 101 healthy controls and the 30 patients with mild and 159 patients with severe COVID-19 had similar plasma adiponectin, which was reduced in the 71 patients with critical disease [18]. The 92 SARS-CoV-2-infected patients, of whom 48% had ARDS, had higher serum adiponectin compared to healthy controls [25]. Thus, previous studies comparing COVID-19 patients with healthy controls have reported different findings, including decreases, increases, and normal levels of serum adiponectin in patient cohorts [18,19,20,21,23,25]. In the current analysis, we observed that serum adiponectin concentrations were similar in healthy controls and patients with moderate COVID-19. Of importance, a reduction in serum adiponectin levels was evident in severely ill patients when compared to those with moderate disease and the control group. This suggests that serum adiponectin levels decrease in critical illness, regardless of SARS-CoV-2 infection. The lower circulating adiponectin levels in patients with severe illness have also been documented in previous studies [38,39]. Our finding is in accordance with the study of Flikweert et al. demonstrating reduced adiponectin levels in critically ill COVID-19 and non-COVID-19 patients [18]. Di Filippo observed no significant differences in adiponectin levels of mild, moderate, and severe COVID-19. Here, 11 not-hospitalized patients were classified as mild, 25 hospitalized patients as moderate and 24 patients admitted to the intensive care unit as severe. This study cohort was comparably small, and the median adiponectin levels of the controls was 4.1 µg/mL, and that of the moderate/severe group was about 20 µg/ml, which is rather high [23]. A comparison of patients with and patients without the need for intensive care revealed similar adiponectin levels in the obese and non-obese cohorts [24]. A problem when comparing different studies is the definition of moderate and severe disease, and in Europe standardized definitions are still lacking [40]. 

We excluded patients with liver cirrhosis from our study as they typically exhibit significantly elevated serum adiponectin levels [31]. In a study by Koch et al., patients with decompensated cirrhosis and sepsis also displayed high adiponectin levels compared to sepsis patients with normal liver function [41]. The inclusion of these cirrhosis patients in their analysis may have hindered the detection of low adiponectin levels in critically ill patients [41]. On the other hand, studies conducted by Perrotta et al. and Flikweert et al. did not involve patients with liver cirrhosis, and they observed low serum adiponectin levels in severe COVID-19 patients compared to healthy controls. Consistent with our findings, these authors reported no significant correlation between adiponectin levels and mortality [18,20]. Additionally, Spirina et al. identified higher adiponectin levels in patients with COVID-19 pneumonia compared to healthy controls, although they did not provide information regarding underlying liver conditions in their patient cohort [25]. 

Serum IL-6 was consistently found to be elevated in severe COVID-19 [42,43,44]. IL-6 is increased in patients with liver cirrhosis [45] and IL-6 was almost 2-fold higher in severe COVID-19 cases compared to moderate cases after excluding patients with liver cirrhosis. A meta-analysis showed almost 3-fold higher serum IL-6 in patients with complicated COVID-19 compared to those with non-complicated disease [32]. The increase in IL-6 in the different study cohorts ranged from 1.5-fold to almost 7-fold, showing that our data are consistent with previous findings [32]. 

There is some evidence that adiponectin exerts protective activities in bacterial infections. Antibacterial response to Gram-positive *Listeria monocytogenes* of adiponectin null mice was severely impaired [46]. Adiponectin was found to induce CC-chemokine ligand 2 in *Listeria monocytogenes*-infected adipocytes, a chemokine important for immune cell recruitment during bacterial infection [47]. Plasma adiponectin reduction has been described as an independent predictor of postoperative infections in individuals following gastric surgery [48]. The influence of bacterial infections on circulating adiponectin levels is relatively understudied. Our current analysis showed that superinfection of COVID-19 patients with bacteria did not result in significant alterations in serum adiponectin levels. It has been shown that endotoxin injection of healthy volunteers did not greatly change plasma adiponectin levels within 24 h [49]. The 27 patients with bacterial infections had strongly increased CRP, whereas adiponectin levels did not change in comparison to the 37 non-infected patients [50]. It seems that bacterial infection does not affect serum adiponectin levels. The current analysis revealed that patients with severe COVID-19 and vancomycin-resistant enterococci had lower serum adiponectin in comparison to severe cases without these infections. Since the first isolation of vancomycin-resistant enterococci in England in 1988, these bacteria have spread rapidly and are now the leading cause of nosocomial infections worldwide. Enterococci have, over time, acquired genes that make them resistant to a variety of antibiotics, including vancomycin [51,52]. Currently, we have no explanation for lower adiponectin levels of patients infected with this Gram-positive bacterium. CRP and procalcitonin levels did not differ between infected and non-infected patients. It remains to be clarified why serum adiponectin levels are low in severe COVID-19 patients, and even lower in severe COVID-19 patients with vancomycin-resistant enterococcal superinfections.

CRP is the most frequently used biomarker for inflammation [53]. Negative correlations of serum adiponectin with CRP were observed in healthy controls, in obesity patients, in patients with metabolic diseases, and in acute dengue patients [54,55,56]. Adiponectin levels did not correlate with CRP in patients with rheumatoid arthritis and patients with Crohn’s disease [57,58]. In beta-thalass emia-major children, positive associations of serum adiponectin with CRP have been described [59]. Similarly, within the moderate COVID-19 cohort, there was a positive association between serum adiponectin and CRP levels. However, in our severe COVID-19 patients, who exhibited elevated CRP levels, there was no correlation between serum adiponectin and CRP. This suggests that the correlations observed between adiponectin levels and CRP, which is a widely used clinical marker for inflammation [60], may be disease-specific and could also be influenced by the severity of the disease. Similarly, serum procalcitonin positively correlated with adiponectin in moderate COVID-19, whereas there were no correlations in severe illness. 

In line with a recent study [61], patients with severe COVID-19 exhibited higher counts of neutrophils, monocytes, and immature granulocytes in their blood compared to those with moderate disease. Interestingly, in the moderate COVID-19 cohort, but not in the severe cohort, there was a negative correlation between adiponectin levels and lymphocyte count. This highlights the variability in associations between serum adiponectin and markers of inflammation based on disease severity. 

Adiponectin levels are elevated in patients with chronic kidney disease [62] and were correspondingly higher in the serum of moderate COVID-19 patients requiring dialysis. In severe cases, the need for dialysis was not associated with higher adiponectin levels, suggesting that severe disease has a greater impact on serum adiponectin levels than renal dysfunction. 

Unexpectedly, there were more patients with hypertension and cardiovascular disease in the moderate COVID-19 cohort than in the severe COVID-19 cohort for an unknown reason, and this is a limitation of our study. Serum adiponectin is low in patients with obesity, hypertension, diabetes, and cardiovascular disease [5,8,14,63]. Such differences were not observed in our patient cohorts. This is most likely because the number of these patients was too small to identify significant differences. A study conducted in Germany reported that nearly 50% of adults aged 50 to 59 years have at least two chronic health conditions, and this number increases with age [64]. 

Although the treatment for SARS-CoV-2 infection was remdesivir and dexamethasone, the medication of individual patients varies due to different comorbidities and disease severity. Common medications, such as statins and metformin, of our cohorts were not documented. Statins have been described to protect against SARS-CoV-2 infection and adverse disease outcomes [65,66,67], and have also been found to induce adiponectin levels [9]. Metformin appears to protect against severe COVID-19 [68] and elevated serum adiponectin levels in several patient cohorts [69]. Thiazolidinediones are oral insulin-sensitizing drugs that increase body weight and circulating adiponectin levels [70] but have no effect on COVID-19 survival [71]. Patients requiring intensive care often receive antibiotics, and this is started early in the course of the disease. Another shortcoming was that, especially in the moderate COVID-19 cohort, not all patients’ data could be collected. The vaccination status of our patients was not documented. This was a single-center study enrolling patients from the Regensburg area, and the relevance of our results to different ethnic groups needs to be confirmed. Controls were from the normal population and did not suffer from morbidities, and laboratory data were not determined. Very large and well-documented multi-center studies are needed to confirm our findings, to improve the generalizability of our study and to account for the effect of common diseases and medications in COVID-19. 

## 5. Conclusions

The current study shows that serum adiponectin levels are reduced in patients with severe COVID-19 compared to moderate cases and controls. Our analysis not only confirms previous findings indicating low adiponectin levels in severe COVID-19, but also suggests that a combination of low adiponectin and high CRP and procalcitonin levels may represent a novel biomarker signature for severe COVID-19. In addition, vancomycin-resistant infections were associated with a further decrease in adiponectin in the severe COVID-19 group, which may be of pathophysiological interest. Further, multi-center studies will be needed to validate adiponectin as an additional biomarker for severe COVID-19 and possibly vancomycin-resistant infections.

## Figures and Tables

**Table 1:** Characteristics of controls, and COVID-19 patients with moderate and severe disease. Median values and minimum and maximum values are listed. In cases where these data were not documented for all patients, the number of patients for whom these data were available is given in superscript. The *p*-values for comparison of moderate and severe COVID-19 are * *p* < 0.05, ** *p* < 0.01, and *** *p* < 0.001, and for comparison of controls and severe COVID-19 ^&&&^ *p* < 0.001 (n = numbers of cells).

Parameter	Moderate COVID-19	Severe COVID-19	Controls
Males/Females	35/29	42/18	14/12
Age (years)	60 (22–83)	57 (31–83)	55 (21–75)
BMI kg/m^2^	26.3 (18.4–42.6) ^32^	29.4 (19.2–66.7) ^56^ **	25.4 (18.0–38.1) ^&&&^
CRP mg/L	26 (0–222)	74 (1–367) ***	not determined
Procalcitonin ng/mL	0.09 (0–24.90)	0.24 (0–25) **	not determined
LDH U/L	224 (127–929) ^39^	378 (162–1534) ***	not determined
AP U/L	96 (38–372) ^29^	99 (37–743)	not determined
Ferritin ng/mL	573 (32–4826) ^45^	1088 (77–21976) *	not determined
IL-6 pg/mL	19 (4–265) ^37^	36 (3–1175)	not determined
TG mg/dL	126 (60–480) ^7^	192 (76–554)	not determined
Lactate mg/dL	10 (4–20) ^6^	10 (4–25)	not determined
Neutrophils n/nL	4.05 (0.13–23.10)	8.18 (0.90–24.91) ***	not determined
Basophils n/nL	0.03 (0–0.21)	0.05 (0.01–0.17) **	not determined
Eosinophils n/nL	0.08 (0–1.19)	0.05 (0–1.07)	not determined
Monocytes n/nL	0.57 (0.07–2.52)	0.71 (0.03–2.21) **	not determined
Lymphocytes n/nL	1.11 (0.09–57.83)	1.20 (0–75.95)	not determined
Immature Granulocytes n/nL	0.03 (0–1.38)	0.25 (0.04–2.92) ***	not determined
Hypertension	22 patients	2 patients ***	0
Diabetes	16 patients	12 patients	0
Cardiovascular disease	12 patients	2 patients **	0
Adiponectin µg/mL	5.2 (1.4–40.0)	2.1 (0.4–13.2) ***	3.5 (1.5–18.5) ^&&&^

**Table 2:** Comparison of serum adiponectin levels of patients with/without dialysis, ventilation, and vasopressor therapy. Patients with liver cirrhosis were excluded. The number of patients treated is given in “N” and the *p*-values are listed.

Intervention/Drug	Moderate COVID-19		Severe COVID-19	
	N	*p*-Value	N	*p*-Value
Dialysis	6	0.052	7	0.573
Ventilation	0	-	58	-
Vasopressor therapy	0	-	40	0.339

**Table 3:** Spearman correlation coefficients (r) and corresponding *p*-values for the correlations of serum adiponectin levels with laboratory values. Patients with liver cirrhosis were excluded (n = numbers of cells).

Laboratory Measures	Moderate COVID-19		Severe COVID-19	
	r	*p*-Value	r	*p*-Value
CRP mg/L	0.299	0.020	−0.048	0.716
Procalcitonin ng/mL	0.462	0.002	0.228	0.082
LDH U/L	0.219	0.200	0.100	0.451
AP U/L	0.190	0.351	−0.142	0.284
Ferritin ng/mL	0.161	0.309	0.194	0.141
IL-6 pg/mL	0.178	0.305	−0.111	0.403
TG mg/dL	0.086 (only 6 patients)	0.872	−0.289	0.038
Lactate mg/dL	0.257 (only 6 patients)	0.624	−0.026	0.847
Neutrophils n/nL	0.046	0.726	0.042	0.753
Basophils n/nL	0.112	0.394	−0.135	0.309
Eosinophils n/nL	0.047	0.291	0.070	0.600
Monocytes n/nL	−0.024	0.724	−0.017	0.899
Lymphocytes n/nL	−0.365	0.004	−0.240	0.067
Immature Granulocytes n/nL	0.118	0.371	−0.125	0.346

## Data Availability

Data supporting reported results can be obtained from the corresponding author.
